# The Influence of Positive and Negative Aspects of Perfectionism on Psychological Distress in Emerging Adulthood: Exploring the Mediating Role of Self-Compassion

**DOI:** 10.3390/bs13110932

**Published:** 2023-11-15

**Authors:** Katerina Koutra, Chrysi Mouatsou, Sofia Psoma

**Affiliations:** Addiction Psychology Laboratory, Department of Psychology, School of Social Sciences, University of Crete, 74100 Rethymnon, Crete, Greece; chrysamouatsou@gmail.com (C.M.); sofpsoma@gmail.com (S.P.)

**Keywords:** emerging adults, perfectionism, psychological distress, self-compassion

## Abstract

Perfectionism constitutes a multidimensional personality trait. According to the diathesis–stress model, perfectionism may predispose individuals to experience increased psychological distress during stressful periods. Since self-compassion is considered as a protective factor within the context of mental health difficulties, the primary objective of this study was to explore the mediating function of self-compassion in the relationship between positive (i.e., high standards and order) and negative (i.e., discrepancy) aspects of perfectionism and psychological distress among Greek emerging adults. The sample consisted of 410 university students (47.6% males and 52.4% females), with a mean age of 20.61 ± 1.88 years. The Almost Perfect Scale-Revised (APS-R), the Self-Compassion Scale (SCS), and the General Health Questionnaire-28 (GHQ-28) were used to assess perfectionism, self-compassion, and psychological distress, respectively. The results indicated that self-compassion mediated the association between positive and negative aspects of perfectionism and different dimensions of psychological distress (i.e., somatic symptoms, anxiety/insomnia, social dysfunction, and severe depression). Specifically, discrepancy and increased levels of high standards were related to lower levels of self-compassion, thus leading to greater endorsement of psychological distress. In contrast, order was associated with higher levels of self-compassion, thus leading to lower levels of psychological distress. The findings of the present study highlight that self-compassion is an overall important area to examine in nonclinical populations. These findings have implications for the effectiveness of cognitive behavioral therapy (CBT) in enhancing individuals’ self-acceptance and awareness of their perfectionistic tendencies, as well as compassion-focused therapy (CFT) in elevating self-compassion, mindfulness, and overall psychological well-being while reducing psychological distress.

## 1. Introduction

Due to societal and demographic changes over the last 50 years, people opt to devote more years to their education and to enter into parenthood at a later age. These changes have resulted in a novel developmental stage, termed “emerging adulthood”, that starts in the late teens and expands through the 20s [1]. This transitional period is characterized by increased levels of exploration in terms of education, career, relationships, and identity, as well as by multiple changes in family roles, living arrangements, and social life [2]. Thus, the instability and uncertainty of this developmental stage constitute major stressors, putting young adults at risk of mental health difficulties. Indeed, various epidemiological studies have shown that mental disorders are highly prevalent among emerging adults, and those suffering from a disorder at this age are more vulnerable to mental problems even 10 years later [3,4].

Perfectionism is a personality trait that refers to setting and striving for extremely high standards, constantly expecting flawless performance and being overly concerned about mistakes [5,6]. Early conceptualizations of perfectionism considered it to be a unidimensional personality disposition, and major contributors in the field have traditionally linked it to maladjustment, as well as to psychological and physical disorders [7,8,9]. However, the current scientific view is more complicated and multifaceted. Slaney et al. [10] distinguished between positive or adaptive perfectionism, which refers to striving for high standards and order, and negative or maladaptive perfectionism, which refers to the feelings of discrepancy between one’s expectations and performance. Several other investigators have also highlighted the difference between adaptive and maladaptive perfectionism, although there is increased heterogeneity regarding the terminology used by researchers. For example, Dunkley et al. [11] considered “personal standards”, referring to the establishment of high expectations and goals for oneself, as the adaptive aspect of perfectionism, and “evaluative concerns”, describing the persistent worries about others’ expectations and the harsh self-judgement, as the maladaptive aspect of perfectionism. Similarly, high personal standards [6] or positive strivings [12] constitute a healthy dimension of perfectionism and are associated with efficacy, goal-oriented strivings, and positive affect. Based on the aforementioned findings, adaptive perfectionists tend to set high standards, are motivated to put effort into achieving their goals, and interpret their faults as opportunities to evolve, while maladaptive perfectionists cannot derive any satisfaction from their accomplishments, and they excessively worry about meeting their own and others’ expectations.

According to the diathesis–stress model, perfectionism may predispose individuals to experience increased psychological distress during stressful periods [13]. Various studies have associated the maladaptive aspect of perfectionism with negative outcomes in an individual’s well-being [14,15]. Regarding emerging adults, a recent meta-analysis found significant associations between perfectionism and symptoms of anxiety, depression, and obsessive–compulsive disorder [16]. In contrast to the positive aspect, only the maladaptive dimension of perfectionism has been correlated with measures of anxiety, stress [17], lower self-esteem, and depressive symptoms [18]. One study supported that high perfectionistic striving, referring to unrealistically high and rigid standards for oneself, and high perfectionistic concerns, referring to self-doubt, overconcern about mistakes, and negative reactions to failure, can predict the levels of negative emotions in college students [19]. Individuals who constantly doubt their abilities tend to be more distressed about their faults, fear possible failures, and consequently, experience higher levels of anxiety during everyday tasks [20].

On the contrary, adaptive perfectionists pursuing high standards and organization tend to have a clearer purpose in life and report higher levels of subjective happiness and well-being [21]. A positive correlation between adaptive perfectionism and psychological well-being has also been found in the study by Kamushadze et al. [22]. In a similar vein, a study in college students supported that adaptive perfectionists demonstrate increased self-esteem, perceived satisfaction, and meaning in life compared to maladaptive perfectionists [23]. Furthermore, perfectionistic strivings, an adaptive dimension of perfectionism, are associated with higher levels of self-efficacy [24]. Finally, it is found that emerging adults who are categorized as adaptive perfectionists have better indices of mental health and mention lower levels of alcohol use [25].

Neff [26] defines self-compassion as the balance between enhanced positive and reduced negative self-responding during times of personal suffering, whether the reason of suffering is failure, inadequacy feelings, or general life challenges. Self-compassion consists of three basic components: kindness, a sense of common humanity, and mindfulness. Kindness is characterized by a sense of caring for and understanding the other person instead of criticizing. The sense of common humanity refers to recognizing that all humans are characterized by imperfections and failures in their everyday life. Finally, being mindful involves facing unpleasant thoughts and feelings and accepting them as they are, without repressing or avoiding them [26,27]. Thus, self-compassion enables people to accept themselves as they are, including their limits and flaws that make them humans. Informally, it is defined as treating oneself with the same care that you would give to a dear friend who is going through a difficult time.

The construct of self-compassion has been investigated in relation to mental health outcomes in various ways. For example, self-compassion may represent characteristics that exist in the parent–child relationship. More specifically, findings show that people who have developed higher levels of anxious or avoidant attachment exhibit lower levels of self-compassion [28,29]. Moreover, early childhood experiences of psychological maltreatment have been correlated with negative effects on well-being during adulthood. Self-esteem and self-compassion mediate this effect, which shows that a negative conception of self-worth and self-competence reduce subjective well-being [30]. Self-compassion is correlated with posttraumatic growth through emotion-focused strategies like positive reframing and acceptance. Emerging adults who use these strategies are more likely to reduce their tendency to ruminate negative thoughts and feel more competent to cope with difficulties [31]. Self-compassion is argued to facilitate better coping strategies and higher levels of mental health. Research conducted on a sample of depressed individuals has shown that self-compassion can be an effective strategy, like reappraisal, to regulate negative emotions [32] and that the ability to tolerate negative thoughts mediates the relationship between self-compassion and depressive symptoms [33]. Stressful situations like failure, rejection, and embarrassment are moderated by self-compassion. More specifically, self-compassion is associated with a way of thinking about these events that is characterized by a lower level of negative emotions and the tendency of people to accept their responsibilities, while at the same time, they ruminate less about their negative experiences and feel fewer negative feelings because of their faults. All in all, self-compassionate people tend to more easily accept their mistakes and undesirable experiences [34].

Taken the aforementioned data together, it could be assumed that maladaptive perfectionists pursue excessively high and unrealistic standards, worry about mistakes, and have a harsh attitude about themselves, while self-compassionate individuals are less self-critical and hold an understanding attitude toward their inadequacies. Although the negative aspect of perfectionism has been linked to adverse effects on psychological health, the exact mechanism that is responsible for this association is not yet fully understood.

Various studies have investigated the potential mediating or moderating role of self-compassion in this relationship. For example, Mehr and Adams [35] supported the idea that perfectionism is associated with depression through self-compassion. Thus, high levels of maladaptive perfectionism may lead to lower levels of self-compassion, which in turn result in the exacerbation of depressive symptoms. In accordance with these results, another study in university students suggested that self-compassion could be a potential variable that explains why maladaptive perfectionists report higher levels of psychological distress compared to adaptive perfectionists [36]. Moreover, the potentially crucial role of self-compassion is highlighted by findings that have supported that self-compassion fully mediates the relationship between perfectionism and subjective well-being in university students [37]. Similarly, Wei et al. [38] confirmed that higher maladaptive perfectionism was linked to higher depression levels through self-compassion in Chinese students, while adaptive perfectionism was associated with increased self-compassion levels, which in turn mitigated the depressive mood. Other studies have also demonstrated that self-compassion could buffer the effects of dysfunctional perfectionism on depressive moods. In other words, individuals who tend to be kinder and less critical toward themselves, even when they are characterized by perfectionistic traits, seem to experience less negative affect [39,40] and lower levels of body image dissatisfaction [41]. Additional evidence has displayed that low levels of self-compassion may act as a susceptibility factor in students with perfectionistic traits, thus leading to burnout, while high self-compassion can buffer the negative effects of perfectionism, potentially protecting individuals from burnout experience [42,43,44].

The critical role of self-compassion is also confirmed by studies with adults having mild symptoms of anxiety and depression [45], as well as with inpatients suffering from depression [46] and individuals with a bipolar disorder diagnosis [47], indicating that self-compassionate individuals are less vulnerable to the negative aspects of perfectionism. Considering all the findings above, self-compassion is regarded as a significant factor that may help perfectionist individuals cope with stress and perfectionistic concerns more effectively, thus diminishing the adverse consequences of perfectionism on psychological well-being.

**Figure 1:**
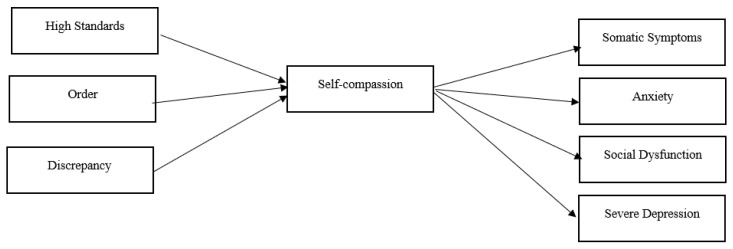
Perfectionism, self-compassion, and psychological distress: hypothesized conceptual model.

The aim of the present study was to investigate the relationship between positive and negative aspects of perfectionism and multiple indicators of psychological distress, as well as the mediating role of self-compassion in the aforementioned associations. To our knowledge, this study is the first that examines the interplay of both the adaptive and maladaptive dimensions of perfectionism with self-compassion and psychological distress in emerging adulthood in Greece. Given that mental health problems are highly prevalent in adolescents and young adults in Greece [48], as they experience multiple life changes and stressors, it is of great importance to shed light on the factors that could alleviate these difficulties. In addition, Greek culture is characterized by both individualism and collectivism [49]; thus, individuals need to set both personal and societal standards, whose interaction may lead to more difficulties and hardships. Consequently, it seems necessary to clarify the relationship between perfectionism and self-compassion in Greek people and how their interplay may promote or aggravate psychological health. Based on the existing literature, it was hypothesized that self-compassion would mediate the relationship between positive and negative aspects of perfectionism and psychological distress. More specifically, we hypothesized that maladaptive characteristics of perfectionism (i.e., discrepancy) would be related to lower levels of self-compassion, thus leading to greater endorsement of psychological distress. In contrast, adaptive characteristics of perfectionism (i.e., high standards and order) would be associated with higher levels of self-compassion, thus leading to lower levels of psychological distress. Figure 1 depicts the hypothesized conceptual model.

## 2. Materials and Methods

### 2.1. Participants

To be eligible for inclusion in the study, individuals needed to meet certain criteria: they had to be students currently enrolled in public universities in Greece, aged between 18 and 25, and possess a proficient comprehension of the Greek language. The sample comprised 410 university students (47.6% males and 52.4% females), with a mean age of Μ = 20.61 years (SD = 1.88 years). Data were gathered from multiple academic departments within Greece, with the majority of participants originating from the field of social sciences, accounting for 53.2% of the total sample. A significant portion of the participants were of Greek nationality (96.1%). Additionally, the vast majority of them reported that they were not in an intimate relationship during the study’s time frame (63.4%). Furthermore, 67.3% of the participants resided alone, while 32.7% were living with others, such as parents, partners, or roommates. It is worth noting that 65.6% of the participants hailed from urban areas, while 34.4% indicated rural origins. Table 1 describes participants’ socio-demographic characteristics.

### 2.2. Measures

#### 2.2.1. Socio-Demographic Background

The questionnaire pertaining to socio-demographic data sought information from participants regarding their gender, age, academic year, department of study, relationship status, place of birth, and details about their current place of residence, including whether they lived alone or with others.

#### 2.2.2. Perfectionism

Perfectionism was assessed using the Almost Perfect Scale-Revised (APS-R) [10], a 23-item instrument designed to evaluate the multifaceted nature of perfectionism across three dimensions: (i) high standards (7 items), which measures personal standards, (ii) order (4 items), which assesses organizational tendencies and the need for order, and (iii) discrepancy (12 items), which gauges the distress stemming from the disparity between performance and one’s standards. Specifically, the discrepancy subscale is typically associated with negative or maladaptive aspects of perfectionism, while the high standards and order subscales are linked to more positive or adaptive aspects. Respondents expressed their agreement with these items using a 7-point Likert-type scale, ranging from 1 (strongly disagree) to 7 (strongly agree). Scores for each subscale were calculated by summing the item scores, with higher scores indicating a greater presence of each dimension of perfectionism. Factor analyses of the APS-R consistently confirmed the structure of these subscales, and the internal consistency reliability for each of the three subscales has been robust, with Cronbach’s coefficient alphas ranging from 0.85 to 0.92 across various samples [10]. The APS-R has been translated and validated for use in the Greek population by Diamantopoulou and Platsidou [50]. In the Greek version, the internal consistency reliability demonstrated satisfactory results (high standards α = 0.78, order α = 0.84, and discrepancy α = 0.90). For the present study, the Cronbach’s alpha coefficients were 0.66 for high standards, 0.73 for order, and 0.91 for discrepancy.

#### 2.2.3. Self-Compassion

Participants’ levels of self-compassion were evaluated using the Self-Compassion Scale (SCS) [26], a comprehensive 26-item instrument designed to measure various facets of global self-compassion. This scale encompasses six distinct subscales, each reflecting a different dimension of self-compassion: self-kindness, self-judgment, common humanity, isolation, mindfulness, and over-identification. Participants rated the frequency with which they typically treat themselves in alignment with the descriptions provided in the items. Items are rated on a 5-point Likert scale, with responses ranging from 1 (almost never) to 5 (almost always). For the negative subscale items, namely self-judgment, isolation, and over-identification, reverse scoring was applied. The scores for all items were then summed to compute a comprehensive self-compassion score. Higher scores on this scale indicate a stronger presence of self-compassion in an individual. Neff [26] reported that the SCS exhibited strong convergent and discriminant validity, as well as robust test–retest reliability (0.93) and internal consistency (0.92). The scale has been adapted and validated for use in the Greek population by Mantzios et al. [51] and Karakasidou et al. [52]. The Greek version of the SCS demonstrated satisfactory internal consistency (α = 0.86) [52]. In the present study, the Cronbach’s alpha coefficient for the SCS was 0.90.

#### 2.2.4. Psychological Distress

To assess participants’ psychological distress, the General Health Questionnaire 28-item version (GHQ-28) was utilized [53]. It is a self-administered questionnaire specifically designed to assess individuals’ overall well-being, as well as their level of psychological distress. The GHQ-28 is divided into four subscales, which evaluate physical symptoms, anxiety/insomnia, social dysfunction, and severe depression. In this study, the Likert scoring system (0, 1, 2, 3) was applied, resulting in a distribution of values that is more amenable, with potential total scores ranging from 0 to 84. Higher scores on this scale indicate a lower level of psychological well-being. The Greek version of the GHQ-28, which employs a Likert response scale, has demonstrated sound psychometric properties (Cronbach’s alpha = 0.90) and recommends a cutoff score of 23/24 to identify individuals at a heightened risk for a mental health condition [54]. For the present study, the Cronbach’s alpha coefficients were as follows: 0.77 for somatic symptoms, 0.79 for anxiety/insomnia, 0.76 for social dysfunction, and 0.86 for severe depression.

### 2.3. Procedure

Participants were recruited either through in-person interaction, where questionnaires were physically distributed on the university campus, or through online channels allowing participants to access a survey via a provided link. Prior to their involvement, participants received a comprehensive explanation regarding the study’s objectives, and they were provided with the researchers’ commitment to maintaining strict anonymity and confidentiality. Engagement in the study was completely voluntary, and there were no adverse consequences for those who chose not to participate or opted to withdraw. Written informed consent was obtained from all participants. Moreover, participants received written instructions for completing the questionnaires and were informed of the estimated time required for the measurements, which was approximately 20 min. All procedures in this study adhered to the ethical standards established by the institutional research committee and were conducted in accordance with the principles outlined in the 1964 Helsinki Declaration and its subsequent amendments or similar ethical standards.

### 2.4. Statistical Analyses

Descriptive statistics were employed to describe the sample’s characteristics. For all continuous variables, means and standard deviations were computed, while for all categorical variables, frequencies and proportions were created. The scales’ reliability was assessed using the Cronbach’s alpha coefficient. Additionally, Pearson’s r correlation coefficient was employed to determine the magnitude of the association between continuous independent and dependent variables. Multivariate associations between measures of perfectionism (high standards, order, and discrepancy) and psychological distress (somatic symptoms, anxiety/insomnia, social dysfunction, and severe depression) through self-compassion (total score) as a mediator were assessed through structural equation modeling, allowing for the estimation of both direct and indirect effects. Bootstrapping was employed with 5000 resamples and 95% confidence intervals (CIs). Significant indirect effects were indicated by CIs that did not contain zero. The chi-square test was used to assess overall model goodness of fit, which was reinforced by comparative fit indices (normed fit index (NFI), comparative fit index (CFI), and absolute fit indices (goodness-of-fit index (GFI))), with values of 0.90 or above indicating a good fit [55,56]. Furthermore, the root mean square of approximation (RMSEA) was taken into account, with values less than 0.08 regarded as acceptable (poor fit > 0.10; moderate fit 0.08–0.10; reasonable fit 0.05–0.08). The estimated direct, indirect, and total effects were presented as standardized regression coefficients and assessed using the corresponding bootstrapped 95% confidence intervals (CI). SPSS Statistics 26 (IBM, Armonk, NY, USA) and AMOS 26 were used for all statistical analyses. All hypothesis testing was conducted with a significance threshold of 0.05 and a two-sided alternative hypothesis.

## 3. Results

### 3.1. Descriptive Results and Bivariate Correlations

Table 2 provides a summary of the means, standard deviations, and the intercorrelations between the primary study variables. The mean scores (±SD) for two out of three APS-R subscales, namely high standards (27.38 ± 3.89) and order (15.41 ± 2.95), were notably high, suggesting a significant presence of perfectionism. The mean scores for all the remaining subscales were within the normal range.

The Pearson’s correlation analyses revealed that self-compassion was significantly correlated with two out of three subscales of APS-R as follows: positively with order and negatively with discrepancy. Furthermore, self-compassion was negatively associated with all dimensions of GHQ-28. Moreover, positive correlations between the discrepancy subscale of APS-R and all subscales of GHQ-28 were found. In addition, significant negative correlations were found between order and somatic symptoms, social dysfunction, and severe depression. The high standards subscale of APS-R was positively related to the anxiety/insomnia subscale of GHQ-28. As far as the dimensions of APS-R are concerned, high standards were positively associated with order. Finally, all subscales of GHQ-28 were significantly correlated between each other.

### 3.2. Mediation Analyses

**Figure 2:**
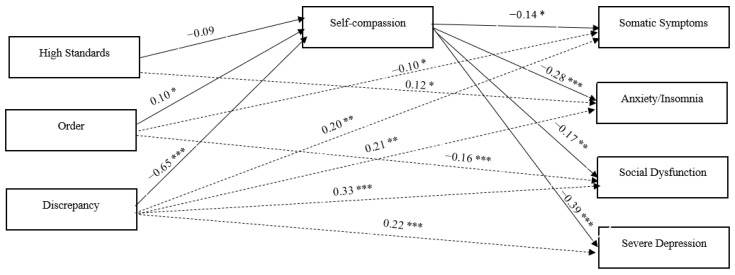
Perfectionism, self-compassion, and psychological distress: standardized path coefficients. **Note**. Dashed lines represent direct effects between X (perfectionism) and Y (psychological distress). Covariances between the three dimensions of X (perfectionism) are not depicted. * *p* < 0.05, ** *p* < 0.01, *** *p* < 0.001.

The full model, encompassing all direct and indirect pathways between the observed variables, including the three dimensions of perfectionism (high standards, order, and discrepancy), self-compassion total score, and the four dimensions of psychological distress (somatic symptoms, anxiety, social dysfunction, and severe depression), was rigorously examined. The results displayed an outstanding fit to the data: chi-square χ^2^ (6) = 0.286 (*p*  =  0.99), GFI  =  0.96, NFI  =  0.96, IFI = 1.00, CFI =  0.96, and RMSEA  =  0.001 (see Figure 2 below).

Table 3 provides an overview of the direct, indirect, and total effects of perfectionism on psychological distress. Notably, there were indications of both direct and indirect effects of the discrepancy subscale of APS-R on all dimensions of psychological distress (somatic symptoms, anxiety/insomnia, social dysfunction, and severe depression) as assessed by GHQ-28, with self-compassion acting as a mediator. Furthermore, the results revealed both a direct and indirect effect of the order subscale of APS-R on somatic symptoms and social dysfunction through self-compassion. However, the order subscale showed only indirect effects via self-compassion on anxiety/insomnia and severe depression. Moreover, the results revealed both a significant direct and indirect effect of high standards on anxiety/insomnia. The high standards subscale showed only indirect effects via self-compassion on somatic symptoms, social dysfunction, and severe depression. Finally, the impact of self-compassion on all dimensions of psychological distress remained substantial, even after accounting for the direct and indirect effects of all other variables within the model.

## 4. Discussion

In this study, we investigated the influence of both the adaptive and maladaptive aspects of perfectionism on the level of psychological distress in Greek emerging adults. The results indicated that both the adaptive and maladaptive dimensions of perfectionism were significantly related, albeit differently, to emerging adults’ psychological distress and that self-compassion mediated the aforementioned relationships. Specifically, we found that maladaptive perfectionism, as indexed by discrepancy levels, positively predicted emerging adults’ psychological distress via low self-compassion. On the contrary, adaptive perfectionism, as indexed by order levels, negatively predicted psychological distress via high self-compassion. Although high standards have been traditionally considered as a positive aspect of perfectionism, in our sample, it was found that increased levels of high standards were negatively related to emerging adults’ self-compassion, further increasing their levels of psychological distress.

This study marks the inaugural exploration of the interconnections between adaptive and maladaptive dimensions of perfectionism, self-compassion, and various facets of psychological distress within the emerging adult population in Greece. The findings of this study align with previous research, confirming that elevated levels of maladaptive perfectionism are associated with a decline in mental well-being, which has been consistently observed in earlier studies [14,15]. Specifically, maladaptive perfectionists experience higher levels of negative affect [19] and anxiety [17,20], suffer more often from depressive symptoms, and report lower self-esteem [18]. Conversely, the adaptive aspect of perfectionism, i.e., striving for high standards and organization, has been found to be associated with better mental health outcomes [25], as well as with higher levels of self-esteem, psychological well-being, and life satisfaction [21,22,23]. Although previous research indicated a negative relationship between adaptive perfectionism and psychological distress, in our study, this was not the case for both adaptive aspects of perfectionism measured. Specifically, we found that order was negatively associated with psychological distress, but high standards were positively related to psychological distress. Therefore, high standards seem to function as a dysfunctional rather than a functional feature of perfectionism in our sample. A possible explanation about this latter finding would be that the responses of most participants in our study were in the high end of the standards subscale of APS-R (77.3% scored between 25 and 49), which indicates perfectionism. Setting high standards and striving for excellence are both positive characteristics, but perfectionism being indicated by extremely high standards is unhealthy since it is worsened by a person’s impression of oneself as continuously flawed or defective. Furthermore, taking into account the mixed perfectionism type in the 2 × 2 model [57,58], it could be possible that positive perfectionism could emerge as a disruptive factor when it co-occurs within individuals alongside negative perfectionism.

The contrasting association of the positive and negative aspects of perfectionism with psychological well-being could be attributed to various mechanisms. For instance, maladaptive perfectionists seem to employ more dysfunctional coping styles, like avoidance, compared to adaptive perfectionists [59]. In addition, negative perfectionism is associated with self-doubt, judgmental attitudes, and fear of failure [11], while positive perfectionism is accompanied by self-efficacy and goal-oriented strivings [6]. Thus, multiple aspects of perfectionistic traits may explain the association with either better or worse mental health outcomes. It is, also, important to note the need for future research to investigate both aspects of perfectionism, as our findings, together with multiple previous data [36,60], highlight the bifactorial nature of the construct and the differential consequences of each perfectionism dimension on psychological distress.

Furthermore, the study findings are consistent with previous research suggesting that higher levels of self-compassion are associated with lower psychopathology. A meta-analysis revealed a large effect size for the relationship between self-compassion and psychopathology, indicating that self-compassion can be a valuable construct for investigating risks and protective factors in mental health [61]. Various studies have proposed the implication of self-compassion in the effective regulation of negative emotions, which may lead to better mental health outcomes [30,31,33]. Consequently, self-compassionate individuals seem to experience fewer psychological difficulties, as they regulate their emotions efficiently, maintain a healthy self-perception, and face life challenges with a positive attitude.

The findings confirmed our hypothesis that self-compassion would mediate the relationship between particular dimensions of perfectionism and psychological distress. More specifically, self-compassion mediated the influence of discrepancy, order, and high standards on somatic symptoms, anxiety/insomnia, social dysfunction, and severe depression. The findings of this study offer support for the idea that self-compassion could be a mechanism by which perfectionism influences the psychological well-being of emerging adults. Our model suggests that maladaptive perfectionists are less self-compassionate, as they tend to harshly judge themselves and set unrealistically high standards, and in turn, the absence of compassionate feelings toward the self may lead to the experience of higher psychological distress. On the other hand, individuals who exhibit adaptive perfectionistic traits related to maintaining order and organization are more self-compassionate while maintaining a caring and understanding relationship with themselves and, as a result, are more protected against psychological distress.

In our study, maladaptive perfectionism (as indexed by discrepancy levels and increased scores in high standards) was found to be associated with lower levels of self-compassion, thus leading to higher levels of psychological distress. In a similar way, Mehr and Adams [35] suggested that self-criticism and feelings of inadequacy, commonly characterizing maladaptive perfectionists, may exacerbate depressive symptoms, while self-compassion could potentially mitigate the impact of negative perfectionism on depression in college students. This buffering effect of self-compassion in the relationship between dysfunctional perfectionism and negative emotions is reported by several studies [39,40,45]. Our findings also suggest that adaptive perfectionism (as indexed by order) is related to higher levels of self-compassion, thus leading to lower levels of psychological distress. Indeed, it is supported that people with adaptive perfectionistic traits tend to exhibit higher self-compassion than people with maladaptive perfectionistic traits [62]. However, there has been limited research on the combined association of adaptive perfectionism and self-compassion with mental health, as most studies focus only on the maladaptive dimension. To our knowledge, only recently has research highlighted the mediating role of self-compassion in the relationship between adaptive perfectionism and psychological functioning [36,38]. Thus, our results not only confirm these findings but also enhance our knowledge regarding the relatively underexplored association of adaptive perfectionistic traits with self-compassion and well-being in a unified model. In conclusion, self-compassion has emerged as a crucial variable for understanding vulnerability and resilience and, more importantly, for understanding the differences between adaptive and maladaptive perfectionists in terms of psychological health.

### 4.1. Strengths and Limitations

The strengths of the present study encompass a substantial sample size, which provides adequate statistical power to detect even minor effects. It also utilized well-established measurement tools and explored numerous variables within integrated models, enhancing our comprehension of the interrelationships between these psychological constructs. Nonetheless, it is crucial to acknowledge several limitations in this study. First, the use of a cross-sectional methodology in the current research precluded making causal inferences, underscoring the need for additional experimental and longitudinal investigations. Although the mediation model we employed featured traits as predictors (perfectionism), attitudes as mediators (self-compassion), and states as dependent variables (past-month psychological distress), this structure minimizes the likelihood of reverse pathways, such as past-month psychological distress causing self-compassion, which in turn affects perfectionism. Second, the study’s reliance on self-administered questionnaires introduces the possibility of response bias and social desirability bias, and this limitation should be noted. Third, about half of the participants come from social sciences backgrounds, which suggests they may have a greater predisposition to compassion in general, potentially influencing the outcomes. Fourth, the indirect effects, particularly those linked to high standards and order, exhibit a relatively modest magnitude. Although our study offers valuable insights into these aspects, their restricted influence should be taken into account when interpreting the findings.

### 4.2. Implications for Research and Clinical Practice

The current study carries significant implications for counseling and mental health services, as it contributes to our understanding of the processes that underlie the link between perfectionism and psychological distress in emerging adults. The study’s findings underscore the importance of investigating self-compassion in nonclinical populations. Furthermore, perfectionism can be targeted as a method to help emerging adults enhance their self-acceptance and self-awareness regarding their perfectionistic tendencies and reduce their vulnerability to mental health challenges. At present, cognitive behavioral therapy (CBT) stands as the primary intervention approach for addressing perfectionism. CBT has been applied and assessed in diverse population groups, encompassing both clinical and nonclinical individuals with elevated levels of perfectionism. For instance, the first randomized controlled trial of group CBT involved a mixed sample characterized by heightened perfectionism and a range of disorders, including anxiety, mood, and eating disorders. This trial affirmed the effectiveness of CBT in reducing perfectionism and psychopathology while simultaneously enhancing self-esteem and overall quality of life [63]. CBT’s effectiveness has also been explored in various student samples, where it has been shown to exert a significant and enduring impact on diminishing perfectionism levels, subsequently leading to reduced levels of anxiety and depression [64,65,66,67].

Although cognitive behavioral therapy (CBT) is widely acknowledged for its effectiveness in reducing perfectionism, our findings also indicate that nurturing self-compassion can serve as a valuable approach in mitigating self-criticism, shame, and negative emotions in individuals with perfectionistic traits, as suggested in other studies [68,69]. The implications regarding the potential effectiveness of compassion-focused therapy (CFT) are also discussed, which can enhance individuals’ self-compassion, mindfulness, and psychological well-being while reducing their stress, anxiety, and depressive symptoms. Recent research supports the idea that even brief programs centered on mindfulness and self-compassion can positively impact students’ mental health by moderating perfectionism [70]. Specifically, interventions rooted in self-compassion have been found to significantly improve various psychosocial outcomes, including rumination, eating attitudes, anxiety, and depression [71]. Notably, Leaviss and Uttley [72] demonstrated that CFT yields multiple therapeutic advantages, especially for individuals characterized by high self-criticism, such as maladaptive perfectionists. Self-compassion could also be a critical intervention target for alleviating depressive and anxiety symptoms, even in bipolar disorder [47].

## 5. Conclusions

In conclusion, the results of the present study underscore significant connections between perfectionism and psychological distress among emerging adults. Nevertheless, it is important to recognize that perfectionism is not a one-dimensional concept; it encompasses both adaptive and maladaptive aspects, which yield varying effects on well-being. Furthermore, our analysis confirmed that self-compassion plays a pivotal role in the dynamic between perfectionism and psychological distress, making it a critical factor in understanding the differing psychological challenges faced by adaptive and maladaptive perfectionists. Psychological interventions aimed at mitigating psychological distress can prove advantageous for emerging adults with perfectionistic tendencies by promoting self-compassion and reducing self-criticism and shame. Future studies should investigate interactions between different aspects of perfectionism, self-compassion, and psychological distress, which might be accomplished by testing moderated mediation models.

## Figures and Tables

**Table 1:** Participants’ socio-demographic characteristics (*n* = 410).

Variable	*Ν*	%
**Gender**		
Male	195	47.6
Female	215	52.4
**Nationality**		
Greek	394	96.1
Other	16	3.9
**Origin**		
Urban	269	65.6
Rural	141	34.4
**Relationship status**		
In a relationship	150	36.6
No relationship	260	63.4
**Year of study**		
1st	75	18.3
2nd	103	25.1
3rd	70	17.1
4th	79	19.3
5th or higher	83	20.2
**Department of study**		
Social sciences	218	53.2
Physical sciences	13	3.2
Life & health sciences	19	4.6
Economics & informatics	151	36.8
Missing	9	2.2
**Living with**		
Alone	276	67.3
With others	134	32.7

**Table 2:** Intercorrelations between the study variables.

	M	SD	1	2	3	4	5	6	7	8
1. High standards (APS-R)	27.38	3.89	1							
2. Order (APS-R)	15.41	2.95	0.29 ***	1						
3. Discrepancy (APS-R)	33.55	9.67	−0.03	−0.07	1					
4. Self-compassion (SCS_total)	79.41	17.59	−0.04	0.12 *	−0.65 ***	1				
5. Somatic symptoms (GHQ-28)	5.55	3.58	0.04	−0.11 *	0.29 ***	−0.28 ***	1			
6. Anxiety/Insomnia (GHQ-28)	6.51	4.25	0.11 *	−0.08	0.40 ***	−0.44 ***	0.63 ***	1		
7. Social dysfunction (GHQ-28)	6.69	3.10	0.03	−0.18 ***	0.45 ***	−0.41 ***	0.42 ***	0.43 ***	1	
8. Severe depression (GHQ-28)	3.15	3.87	0.07	−0.13 **	0.48 ***	−0.55 ***	0.36 ***	0.47 ***	0.45 ***	1

Abbreviations: APS-R: Almost Perfect Scale-Revised; GHQ-28: General Health Questionnaire-28; SCS: Self-Compassion Scale. * *p* < 0.05, ** *p* < 0.01, *** *p* < 0.001.

**Table 3:** Direct, indirect, and total effects of perfectionism on psychological distress through self-compassion.

	Direct Effect ^a^			Indirect Effect ^a^			Total Effect ^a^		
	*β*	95% CI	*p*	*β*	95% CI	*p*	*β*	95% CI	*p*
High standards → Self-compassion	−0.09	(−0.19, 0.01)	0.057	**-**	**-**	**-**	−0.09	(−0.19, 0.01)	0.057
High standards → Somatic symptoms	0.07	(−0.03, 0.17)	0.190	**0.01**	**(0.00, 0.04)**	**0.043**	0.08	(−0.02, 0.19	0.117
High standards → Anxiety/Insomnia	**0.12**	**(0.02, 0.22)**	**0.020**	**0.03**	**(0.00, 0.06)**	**0.035**	**0.15**	**(0.05, 0.24)**	**0.004**
High standards → Social dysfunction	0.08	(−0.02, 0.18)	0.102	**0.02**	**(0.00, 0.04)**	**0.026**	0.10	(−0.00, 0.20)	0.056
High standards → Severe depression	0.08	(−0.02, 0.18)	0.127	**0.04**	**(0.00, 0.08)**	**0.043**	0.12	(−0.01, 0.23)	0.064
Order → Self-compassion	**0.10**	**(0.02, 0.20)**	**0.014**	**-**	**-**	**-**	**0.10**	**(0.02, 0.20)**	**0.014**
Order → Somatic symptoms	**−0.10**	**(−0.19, −0.01)**	**0.041**	**−0.01**	**(−0.05, −0.00)**	**0.021**	**−0.11**	**(−0.21, −0.02)**	**0.024**
Order → Anxiety/Insomnia	−0.06	(−0.17, 0.33)	0.191	**−0.03**	**(−0.07, −0.01)**	**0.009**	−0.09	(−0.20, 0.01)	0.060
Order → Social dysfunction	**−0.16**	**(−0.24, −0.07)**	**<0.001**	**−0.02**	**(−0.04, −0.00)**	**0.009**	**−0.17**	**(−0.26, −0.09)**	**<0.001**
Order → Severe depression	−0.09	(−0.19, 0.01)	0.080	**−0.04**	**(−0.08, −0.01)**	**0.010**	**−0.13**	**(−0.24, −0.02)**	**0.013**
Discrepancy → Self-compassion	**−0.65**	**(−0.71, −0.58)**	**<0.001**	**-**	**-**	**-**	**−0.65**	**(−0.71, −0.58)**	**<0.001**
Discrepancy → Somatic symptoms	**0.20**	**(0.06, 0.32)**	**0.004**	**0.09**	**(0.01, 0.18)**	**0.030**	**0.29**	**(0.19, 0.39)**	**<0.001**
Discrepancy → Anxiety/Insomnia	**0.21**	**(0.09, 0.35)**	**0.001**	**0.18**	**(0.11, 0.26)**	**<0.001**	**0.40**	**(0.30, 0.49)**	**<0.001**
Discrepancy → Social dysfunction	**0.33**	**(0.23, 0.44)**	**<0.001**	**0.11**	**(0.03, 0.18)**	**0.005**	**0.44**	**(0.36, 0.52)**	**<0.001**
Discrepancy → Severe depression	**0.22**	**(0.11, 0.33)**	**<0.001**	**0.25**	**(0.17, 0.33)**	**<0.001**	**0.48**	**(0.39, 0.55)**	**<0.001**
Self-compassion → Somatic symptoms	**−0.14**	**(−0.28, −0.01)**	**0.031**	**-**	**-**	**-**	**−0.14**	**(−0.28, −0.01)**	**0.031**
Self-compassion → Anxiety/Insomnia	**−0.28**	**(−0.40, −0.16)**	**<0.001**	**-**	**-**	**-**	**−0.28**	**(−0.40, −0.16)**	**<0.001**
Self-compassion → Social dysfunction	**−0.17**	**(−0.28, −0.05)**	**0.006**	**-**	**-**	**-**	**−0.17**	**(−0.28, −0.05)**	**0.006**
Self-compassion → Severe depression	**−0.39**	**(−0.50, −0.26)**	**<0.001**	**-**	**-**	**-**	**−0.39**	**(−0.50, −0.26)**	**<0.001**

Abbreviations: model fit indices: chi-square χ^2^ (6) = 0.286 (*p*  =  0.99), GFI  =  0.96, NFI  =  0.96, IFI = 1.00, CFI =  0.96, RMSEA  =  0.001. **^a^** Standardized regression coefficients, corresponding bootstrapped 95% confidence intervals and associated *p* values. Bold font indicates significant effects (*p* < 0.05).

## Data Availability

The data that support the findings of this study are available from the corresponding author upon reasonable request.

## References

[1] Arnett J.J. (2000). Emerging Adulthood: A Theory of Development from the Late Teens through the Twenties. Am. Psychol..

[2] Arnett J.J., Žukauskienė R., Sugimura K. (2014). The New Life Stage of Emerging Adulthood at Ages 18–29 Years: Implications for Mental Health. Lancet Psychiatry.

[3] Auerbach R.P., Mortier P., Bruffaerts R., Alonso J., Benjet C., Cuijpers P., Demyttenaere K., Ebert D.D., Green J.G., Hasking P. (2018). WHO World Mental Health Surveys International College Student Project: Prevalence and Distribution of Mental Disorders. J. Abnorm. Psychol..

[4] Gustavson K., Knudsen A.K., Nesvåg R., Knudsen G.P., Vollset S.E., Reichborn-Kjennerud T. (2018). Prevalence and Stability of Mental Disorders among Young Adults: Findings from a Longitudinal Study. BMC Psychiatry.

[5] Flett G.L., Hewitt P.L., Flett G.L., Hewitt P.L. (2002). Perfectionism and Maladjustment: An Overview of Theoretical, Definitional, and Treatment Issues. Perfectionism: Theory, Research, and Treatment.

[6] Frost R.O., Marten P., Lahart C., Rosenblate R. (1990). The Dimensions of Perfectionism. Cogn. Ther. Res..

[7] Burns D.D. (1980). The Perfectionist’s Script for Self-Defeat.

[8] Hollender M.H. (1965). Perfectionism. Compr. Psychiatry.

[9] Pacht A.R. (1984). Reflections on Perfection. Am. Psychol..

[10] Slaney R.B., Rice K.G., Mobley M., Trippi J., Ashby J.S. (2001). The Revised Almost Perfect Scale. Meas. Eval. Couns. Dev..

[11] Dunkley D.M., Blankstein K.R., Masheb R.M., Grilo C.M. (2006). Personal Standards and Evaluative Concerns Dimensions of “Clinical” Perfectionism: A Reply to Shafran et al. (2002, 2003) and Hewitt et al. (2003). Behav. Res. Ther..

[12] Frost R.O., Heimberg R.G., Holt C.S., Mattia J.I., Neubauer A.L. (1993). A Comparison of Two Measures of Perfectionism. Personal. Individ. Differ..

[13] Dunkley D.M., Zuroff D.C., Blankstein K.R. (2003). Self-critical perfectionism and daily affect: Dispositional and situational influences on stress and coping. J. Pers. Soc. Psychol..

[14] DiBartolo P.M., Li C.Y., Frost R.O. (2008). How Do the Dimensions of Perfectionism Relate to Mental Health?. Cogn. Ther. Res..

[15] Limburg K., Watson H.J., Hagger M.S., Egan S.J. (2017). The Relationship between Perfectionism and Psychopathology: A Meta-analysis. J. Clin. Psychol..

[16] Lunn J., Greene D., Callaghan T., Egan S.J. (2023). Associations between Perfectionism and Symptoms of Anxiety, Obsessive-Compulsive Disorder and Depression in Young People: A Meta-Analysis. Cogn. Behav. Ther..

[17] Bieling P.J., Israeli A.L., Antony M.M. (2004). Is Perfectionism Good, Bad, or Both? Examining Models of the Perfectionism Construct. Personal. Individ. Differ..

[18] Ashby J.S., Rice K.G., Martin J.L. (2006). Perfectionism, Shame, and Depressive Symptoms. J. Couns. Dev..

[19] Smith M.M., Saklofske D.H., Yan G., Sherry S.B. (2015). Perfectionistic Strivings and Perfectionistic Concerns Interact to Predict Negative Emotionality: Support for the Tripartite Model of Perfectionism in Canadian and Chinese University Students. Personal. Individ. Differ..

[20] Smith M.M., Vidovic V., Sherry S.B., Stewart S.H., Saklofske D.H. (2017). Are Perfectionism Dimensions Risk Factors for Anxiety Symptoms? A Meta-Analysis of 11 Longitudinal Studies. Anxiety Stress Coping.

[21] Suh H., Gnilka P.B., Rice K.G. (2017). Perfectionism and Well-Being: A Positive Psychology Framework. Personal. Individ. Differ..

[22] Kamushadze T., Martskvishvili K., Mestvirishvili M., Odilavadze M. (2021). Does Perfectionism Lead to Well-Being? The Role of Flow and Personality Traits. Eur. J. Psychol..

[23] Park H.J., Jeong D.Y. (2015). Psychological Well-Being, Life Satisfaction, and Self-Esteem among Adaptive Perfectionists, Maladaptive Perfectionists, and Nonperfectionists. Personal. Individ. Differ..

[24] Stoeber J., Hutchfield J., Wood K.V. (2008). Perfectionism, Self-Efficacy, and Aspiration Level: Differential Effects of Perfectionistic Striving and Self-Criticism after Success and Failure. Personal. Individ. Differ..

[25] Nelsen S.K., Kayaalp A., Page K.J. (2021). Perfectionism, Substance Use, and Mental Health in College Students: A Longitudinal Analysis. J. Am. Coll. Health.

[26] Neff K.D. (2003). The Development and Validation of a Scale to Measure Self-Compassion. Self Identity.

[27] Neff K.D., Germer C.K. (2013). A pilot study and randomized controlled trial of the mindful self-compassion program. J. Clin. Psychol..

[28] Neff K.D., McGehee P. (2010). Self-Compassion and Psychological Resilience among Adolescents and Young Adults. Self Identity.

[29] Raque-Bogdan T.L., Ericson S.K., Jackson J., Martin H.M., Bryan N.A. (2011). Attachment and Mental and Physical Health: Self-Compassion and Mattering as Mediators. J. Couns. Psychol..

[30] Wu Q., Cao H., Lin X., Zhou N., Chi P. (2022). Child Maltreatment and Subjective Well-Being in Chinese Emerging Adults: A Process Model Involving Self-Esteem and Self-Compassion. J. Interpers. Violence.

[31] Munroe M., Al-Refae M., Chan H.W., Ferrari M. (2022). Using Self-Compassion to Grow in the Face of Trauma: The Role of Positive Reframing and Problem-Focused Coping Strategies. Psychol. Trauma Theory Res. Pract. Policy.

[32] Diedrich A., Grant M., Hofmann S.G., Hiller W., Berking M. (2014). Self-Compassion as an Emotion Regulation Strategy in Major Depressive Disorder. Behav. Res. Ther..

[33] Diedrich A., Burger J., Kirchner M., Berking M. (2017). Adaptive Emotion Regulation Mediates the Relationship between Self-compassion and Depression in Individuals with Unipolar Depression. Psychol. Psychother. Theory Res. Pract..

[34] Leary M.R., Tate E.B., Adams C.E., Batts Allen A., Hancock J. (2007). Self-Compassion and Reactions to Unpleasant Self-Relevant Events: The Implications of Treating Oneself Kindly. J. Pers. Soc. Psychol..

[35] Mehr K.E., Adams A.C. (2016). Self-Compassion as a Mediator of Maladaptive Perfectionism and Depressive Symptoms in College Students. J. Coll. Stud. Psychother..

[36] Kawamoto A., Sheth R., Yang M., Demps L., Sevig T. (2023). The Role of Self-Compassion Among Adaptive and Maladaptive Perfectionists in University Students. Couns. Psychol..

[37] Stoeber J., Lalova A.V., Lumley E.J. (2020). Perfectionism, (Self-) Compassion, and Subjective Well-Being: A Mediation Model. Personal. Individ. Differ..

[38] Wei S., Li L., Shi J., Liang H., Yang X. (2021). Self-Compassion Mediates the Perfectionism and Depression Link on Chinese Undergraduates. Ann. Palliat. Med..

[39] Ferrari M., Yap K., Scott N., Einstein D.A., Ciarrochi J. (2018). Self-Compassion Moderates the Perfectionism and Depression Link in Both Adolescence and Adulthood. PLoS ONE.

[40] Rocha L.F.D.D., Falcone E.M.D.O., Hernandez J.A.E. (2022). Does self-compassion mediate the relation between perfectionism and psychopathological outcomes?. Psicol. Clínica.

[41] Barnett M.D., Sharp K.J. (2016). Maladaptive Perfectionism, Body Image Satisfaction, and Disordered Eating Behaviors among US College Women: The Mediating Role of Self-Compassion. Personal. Individ. Differ..

[42] Cabaços C., Macedo A., Carneiro M., Brito M.J., Amaral A.P., Araújo A., Pereira A.T. (2023). The Mediating Role of Self-Compassion and Repetitive Negative Thinking in the Relationship between Perfectionism and Burnout in Health-Field Students: A Prospective Study. Personal. Individ. Differ..

[43] Pereira A.T., Brito M.J., Cabaços C., Carneiro M., Carvalho F., Manão A., Macedo A. (2022). The Protective Role of Self-Compassion in the Relationship between Perfectionism and Burnout in Portuguese Medicine and Dentistry Students. Int. J. Environ. Res. Public Health.

[44] Richardson C.M., Trusty W.T., George K.A. (2020). Trainee Wellness: Self-Critical Perfectionism, Self-Compassion, Depression, and Burnout among Doctoral Trainees in Psychology. Couns. Psychol. Q..

[45] Adams V., Howell J., Egan S.J. (2022). Self-Compassion as a Moderator between Clinical Perfectionism and Psychological Distress. Aust. Psychol..

[46] Abdollahi A., Allen K.A., Taheri A. (2020). Moderating the Role of Self-Compassion in the Relationship between Perfectionism and Depression. J. Ration. Emotive Cogn. Behav. Ther..

[47] Fletcher K., Yang Y., Johnson S.L., Berk M., Perich T., Cotton S., Murray G. (2019). Buffering against Maladaptive Perfectionism in Bipolar Disorder: The Role of Self-Compassion. J. Affect. Disord..

[48] Basta M., Micheli K., Koutra K., Fountoulaki M., Dafermos V., Drakaki M., Faloutsos K., Soumaki E., Anagnostopoulos D., Papadakis N. (2022). Depression and Anxiety Symptoms in Adolescents and Young Adults in Greece: Prevalence and Associated Factors. J. Affect. Disord. Rep..

[49] Nezlek J.B., Kafetsios K., Smith C.V. (2008). Emotions in Everyday Social Encounters: Correspondence Between Culture and Self-Construal. J. Cross-Cult. Psychol..

[50] Diamantopoulou G., Platsidou M. (2014). Factorial Validity and Psychometric Properties of the Greek Version of the Almost Perfect Scale Revised (APS-R). Hell. J. Psychol..

[51] Mantzios M., Wilson J.C., Giannou K. (2015). Psychometric Properties of the Greek Versions of the Self-Compassion and Mindful Attention and Awareness Scales. Mindfulness.

[52] Karakasidou E., Pezirkianidis C., Galanakis M., Stalikas A. (2017). Validity, Reliability and Factorial Structure of the Self Compassion Scale in the Greek Population. J. Psychol. Psychother..

[53] Goldberg D.P., Gater R., Sartorius N., Ustun T.B., Piccinelli M., Gureje O., Rutter C. (1997). The Validity of Two Versions of the GHQ in the WHO Study of Mental Illness in General Health Care. Psychol. Med..

[54] Garyfallos G., Karastergiou A., Adamopoulou A., Moutzoukis C., Alagiozidou E., Mala D., Garyfallos A. (1991). Greek Version of the General Health Questionnaire: Accuracy of Translation and Validity. Acta Psychiatr. Scand..

[55] Byrne B.M. (2010). Structural Equation Modeling with AMOS: Basic Concepts, Applications, and Programming.

[56] Hair J.F., Anderson R.E., Tatham R.L., Black W.C. (1998). Multivariate Data Analysis.

[57] Gaudreau P. (2019). On the Distinction between Personal Standards Perfectionism and Excellencism: A Theory Elaboration and Research Agenda. Perspect. Psychol. Sci..

[58] Gaudreau P., Thompson A. (2010). Testing a 2 × 2 Model of Dispositional Perfectionism. Personal. Individ. Differ..

[59] Abdollahi A., Hosseinian S., Asmundson G.J. (2018). Coping Styles Mediate Perfectionism Associations with Depression among Undergraduate Students. J. Gen. Psychol..

[60] Hill R.W., Huelsman T.J., Araujo G. (2010). Perfectionistic Concerns Suppress Associations between Perfectionistic Strivings and Positive Life Outcomes. Personal. Individ. Differ..

[61] MacBeth A., Gumley A. (2012). Exploring Compassion: A Meta-Analysis of the Association between Self-Compassion and Psychopathology. Clin. Psychol. Rev..

[62] Sahin E.E. (2021). Self-Compassion as a Mediator Between Perfectionism and Life-Satisfaction Among University Students. Int. J. Prog. Educ..

[63] Handley A.K., Egan S.J., Kane R.T., Rees C.S. (2015). A Randomised Controlled Trial of Group Cognitive Behavioural Therapy for Perfectionism. Behav. Res. Ther..

[64] Arana F.G., Miracco M.C., Galarregui M.S., Keegan E.G. (2017). A Brief Cognitive Behavioural Intervention for Maladaptive Perfectionism in Students: A Pilot Study. Behav. Cogn. Psychother..

[65] Arpin-Cribbie C., Irvine J., Ritvo P. (2012). Web-Based Cognitive-Behavioral Therapy for Perfectionism: A Randomized Controlled Trial. Psychother. Res..

[66] Chand S.P., Chibnall J.T., Slavin S.J. (2018). Cognitive Behavioral Therapy for Maladaptive Perfectionism in Medical Students: A Preliminary Investigation. Acad. Psychiatry.

[67] Radhu N., Daskalakis Z.J., Arpin-Cribbie C.A., Irvine J., Ritvo P. (2012). Evaluating a Web-Based Cognitive-Behavioral Therapy for Maladaptive Perfectionism in University Students. J. Am. Coll. Health.

[68] Cheli S., Cavalletti V., Flett G.L., Hewitt P.L. (2022). Perfectionism Unbound: An Integrated Individual and Group Intervention for Those Hiding Imperfections. J. Clin. Psychol..

[69] Matos M., Steindl S.R. (2020). “You Are Already All You Need to Be”: A Case Illustration of Compassion-focused Therapy for Shame and Perfectionism. J. Clin. Psychol..

[70] Woodfin V., Molde H., Dundas I., Binder P.E. (2021). A randomized control trial of a brief self-compassion intervention for perfectionism, anxiety, depression, and body image. Front. Psychol..

[71] Ferrari M., Hunt C., Harrysunker A., Abbott M.J., Beath A.P., Einstein D.A. (2019). Self-Compassion Interventions and Psychosocial Outcomes: A Meta-Analysis of RCTs. Mindfulness.

[72] Leaviss J., Uttley L. (2015). Psychotherapeutic Benefits of Compassion-Focused Therapy: An Early Systematic Review. Psychol. Med..

